# Recent progress in actuation technologies of micro/nanorobots

**DOI:** 10.3762/bjnano.12.59

**Published:** 2021-07-20

**Authors:** Ke Xu, Bing Liu

**Affiliations:** 1 School of Information & Control Engineering, Shenyang Jianzhu University, Shenyang 110168, China

**Keywords:** actuation methods, external field actuation, micro/nanorobots, self-actuation

## Abstract

As a research field of robotics, micro/nanorobots have been extensively studied in recent years because of their important application prospects in biomedical fields, such as medical diagnosis, nanoscale surgery, and targeted therapy. In this article, recent progress on micro/nanorobots is reviewed regarding actuation technologies. First, the different actuation mechanisms are divided into two types, external field actuation and self-actuation. Then, a few latest achievements on actuation methods are presented. On this basis, the principles of various actuation methods and their limitations are also analyzed. Finally, some key challenges in the development of micro/nanorobots are summarized and the next development direction of the field is explored.

## Introduction

In the past few decades, micro/nanorobots have developed rapidly as an emerging field of robotics. Macroscale robots are limited in their use in specific scenarios such as working in small spaces. In contrast, micro/nanorobots can operate non-invasively in small, inaccessible spaces and play an important role in biomedicine and other fields, such as targeted drug delivery to treat cancer [1–6], nanosurgery [7–8], and environmental treatment [9–11]. In 1959, Feynman [12] first proposed the idea of using microrobots in medical treatment. It has been envisioned that nanorobots could be implanted into human bodies to self-replicate and kill various viruses. Scientists have been more and more enthusiastic about the research on micro/nanorobots. The combination of micro/nanorobots with biomedicine can solve problems that cannot be solved by traditional medicine, and is expected to bring up a new medical revolution [13]. Therefore, scientists continue to explore and study micro/nanorobotics technology. However, due to the small size of micro/nanorobots, they are difficult to design, build, and navigate. Some traditional robotic theories and technologies cannot be applied in micro/nanorobots. When the size of an object drops to the micro/nanoscale, the micro/nanorobot movement in fluids is in the regime of low Reynolds numbers [14–15]. In this case, the viscous force is dominant, and the inertial force is negligible. Hence, micro/nanorobots must be continuously powered for actuation. However, due to the tiny size, power sources such as batteries and engines are difficult to be loaded on micro/nanorobots. Therefore, actuation technologies have been a core content of research in the field of micro/nanorobots [16].

One way to actuate micro/nanorobots to achieve motion is to transform energy. Micro/nanorobots can convert magnetic energy, light energy, acoustic energy, or other forms of energy into kinetic energy or actuation force, so as to perform work tasks flexibly and efficiently at the micro/nanoscale. According to the different actuation mechanisms, actuation technologies that have already been applied to micro/nanorobots can be summarized into two categories. One is external field actuation, including external magnetic fields, electric fields, light fields, and acoustic fields. The other is self-actuation, which includes chemical self-actuation, biological self-actuation, and other methods. Various actuation methods are used for micro/nanorobots, which add a certain degree of complexity compared to traditional macroscale robots. The selection of different actuation methods for different micro/nanorobots will directly affect motion, so the research on actuation technologies of micro/nanorobots is very important and profound.

This paper aims to review the research progress of micro/nanorobots from two aspects: external field actuation and self-actuation. The application of different actuation methods in micro/nanorobots is described, and principles and characteristics are introduced in detail. In addition, potential applications and possible challenges in the current development are also presented. In the end, future actuation methods for micro/nanorobots are explored.

## Review

### External field actuation

#### Magnetic field actuation

In recent years, the development of magnetic field-actuated micro/nanorobots has become increasingly mature. Back in 2005, Dreyfus et al. [17] created a micro/nanorobot actuated by a variant magnetic field. By applying a constant magnetic field along the horizontal direction of the robot and an oscillating magnetic field perpendicular to the robot, the robot can make a motion similar to the motion of cilia in microorganisms. The success of this research laid the foundation for future research.

Steager et al. [18] proposed a magnetically actuated robotic system capable of fully automated manipulation of cells and microbeads and prepared a magnetic U-shaped robot, which was actuated with five electromagnetic coil controllers to generate a gradient magnetic field. In order to prepare a magnetic U-shaped robot, magnetic nanoparticles and photoresist are uniformly mixed and a U-shaped pattern is processed by photolithography. The robot could capture and automatically transport microbeads injected with chemicals to specific locations in neurons under the control of a gradient magnetic field, which has potential applications in targeted drug delivery.

Li et al. [19] designed a fish-like magnetic actuation micro/nanorobot with a passive gold segment as the head, two active nickel segments as the body, and one passive gold segment as the caudal fin, all connected by a flexible structure of porous silver. The swimming mode of the robot under the action of a plane oscillating magnetic field is very similar to the propulsion mode of fish. Therefore, the robot has high propulsion efficiency and can achieve quite high speeds. By using an oscillating magnetic field with a frequency of 11 Hz, a maximum speed of 30.9 μm/s, corresponding to a dimensionless speed of 0.6, has been obtained.

After completing the above design, Li et al. [20] worked on the development of a freestyle swimmer-type of magnetically actuated micro/nanorobots, namely a symmetric multi-linked two-arm nanoswimmer, which consists of a central gold body segment and two nickel arm segments connected by flexible porous silver hinges. The flexible porous silver segment makes the robot swing symmetrically. By magnetizing the nickel segment, the two arms of the robot can swing in different directions under the action of the oscillating magnetic field, realizing a freestyle swimming mode. The speed and orientation of the robot can be adjusted remotely by adjusting the magnetic field. The maximum speed is 59.6 μm/s, which is equivalent to a relative speed of about twelve body lengths per second. For the first time, oscillating magnetic fields were used to generate a non-planar swinging swimming mode. The fish-like micro/nanorobot and the freestyle swimmer micro/nanorobot both have obvious advantages in terms of maximum movement speed and propulsion efficiency with the latter robot performing better.

Mushtaq et al. [21] designed a soft hybrid nanorobot that mimics an electric eel, called nanoeel. It has a smart flexible tail, made of polyvinylidene fluoride copolymer, connected to the head of a polypyrrole nanowire. The head is decorated with a nickel ring for magnetic actuation. When an alternating magnetic field is applied, the magnetic head module (nickel–polypyrrole) oscillates, and then, due to the piezoelectric effect, the ferroelectric tail bends and the electric polarization changes. Experimental analysis shows that changing the magnitude and frequency of the magnetic field can transform the motion of the nanoeel from surface walking to three-dimensional swing and spiral motion. At the same time, by applying a suitable magnetic field, the nanoeel can be accurately guided to a target location for drug release. Under a magnetic field of 10 mT and 7 Hz, drug delivery to cancer cells could be achieved. The efficiency of killing cancer cells is 35% in the drug release mode and 10% in the swimming mode. The results of this research quantitatively confirmed the ability of using nanorobots for targeted delivery of medicines. This will promote the use of nanorobots in clinical practice and provide a solid foundation for future research.

For the treatment of cancer, Wang et al. [22] developed a new type of micro/nanorobot, which can observe the state of cancer. This robot uses multi-stage magnetic tweezers for manipulation, which can precisely move in living cells. The multi-stage magnetic tweezers are composed of six magnetic poles and six coils, installed at the top and the bottom, respectively. The tip is made of foil with high permeability, which ensures a big magnetic gradient. The device was placed on a glass slide of a microscope to ensure that the robot is exposed to the force of a three-dimensional magnetic field when entering cells placed on the glass slide. The use of robots to measure the force between cells may become a means of detecting cancer in its early stage. This research brings new hope for cancer treatment.

Kim et al. [23] designed a micro/nanorobot magnetically actuated in three dimensions, which can accurately transport cultured nerve cells to connect disconnected nerve clusters. It can guide the direction of axon growth and selectively reconstruct neural networks in vitro. The micro/nanorobot can be used as a basis for the targeted delivery of nerve cells and the formation of active neural networks in vitro, thereby promoting the research of neural networks and neural connectivity, offering reproducibility, selectivity, and precise connection. This provides a potential platform for advanced in vitro controllable models of artificial neural networks.

With the research on single magnetic field-driven micro/nanorobots progressing, scholars hope to explore the operation of groups of micro/nanorobots. Due to their small size and low cost, tens to hundreds of micro/nanorobots can work in parallel to perform tasks that are too cumbersome and impossible for macroscale robots. Hsu et al. [24] designed a control platform to study the behavior of large groups, including a scalable system and a modular circuit architecture. It was used to coordinate up to 16 micro/nanorobots for minimally invasive, collision-free two-dimensional position control. Experiments show that the maximum can reach 288 degrees of freedom, and the introduction of random jitter can achieve a 100% success rate. Therefore, it can be said that swarm control will become a hot topic of micro/nanorobot research.

**Figure 1:**
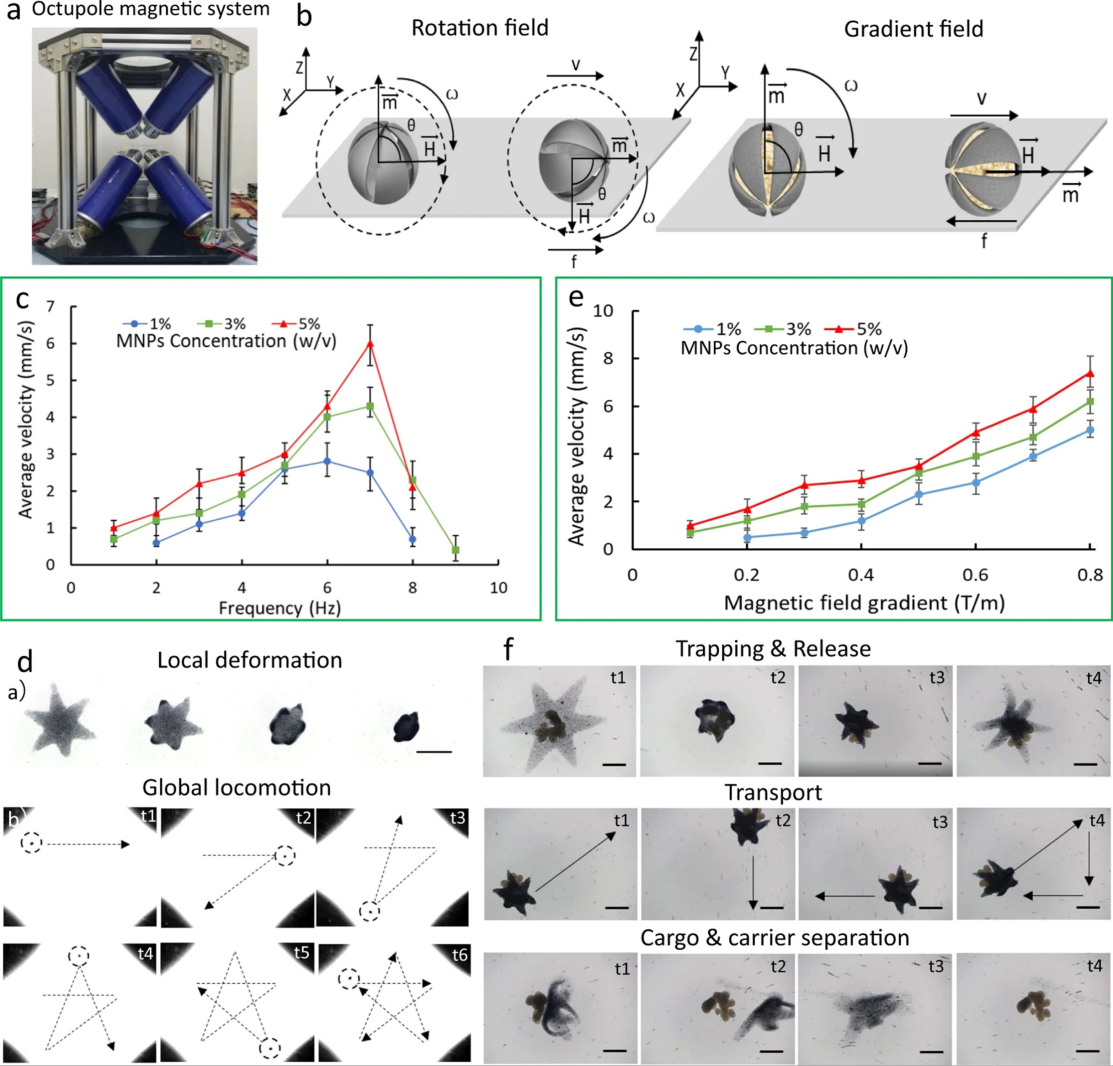
(a) Octupole magnetic system used in the experiment. (b) Schematic illustration of the magnetic actuation of an ionic shape-morphing microrobotic end-effector (ISME) under a rotating field and a gradient field; *V* represents the translational velocity, *H* denotes the strength of the magnetic field, *m* indicates the direction of magnetization of the ISME, *f* represents the force of friction, ω indicates the rotation velocity of the microsphere, and θ denotes the tilt angle between the direction of the magnetic field and the direction of magnetization of the ISME. (c) Velocity–frequency profiles for ISMEs with different MNP concentrations (1%, 3%, and 5%) under a rotating magnetic field. Each dot represents the average velocity for five different ISMEs with hexagram shapes ± standard error of the mean (s.e.m.). (d) a) Images of the ISME with encapsulated MNPs during folding in response to a pH change. Scale bar: 500 µm. b) Time series of the rotating-field-controlled locomotion of an ISME with time instants marked and motion trajectories delineated. Scale bar: 4 mm. (e) Velocity–frequency profiles of ISMEs with different MNP concentrations (1%, 3%, and 5%) under a gradient magnetic field. Each dot represents the average velocity for five different ISMEs with hexagram shapes ± s.e.m. (f) Images of MNP-loaded ISMEs performing capture, transportation, and release of cell aggregates, separation following release, and eventually dissolution in response to a change of the pH value. Scale bar: 250 µm. Reproduced from [25], Copyright © 2021 Zheng et al., licensed under a Creative Commons Attribution 4.0 International License, http://creativecommons.org/licenses/by/4.0/.

Inspired by the movement process of starfish preying on shellfish, Zheng et al. [25] designed a sea star-like microrobot, the flexible tentacles of which can effectively conform to the outer contours of any target with autonomous deformation in a liquid environment for grasping and releasing. The robot has multiple actuation modes, for example, through trapping of magnetic microspheres or through encapsulating magnetic nanomaterials in the robot body, as shown in Figure 1. After reaching the target area, different tasks such as attaching, delivering, and sampling are completed through autonomous deformation and adjustment of the posture. This achievement is the first to use a biodegradable biomaterial to realize self-deformation of microrobots under environmental perception, thereby solving the problem of integrated operation of microrobots in a closed living environment. This application in minimally invasive medicine, soft robotics, and smart materials has attracted more and more attention.

#### Electric field actuation

External electric fields can be used to actuate micro/nanorobots. Under normal circumstances, electric fields and magnetic fields are inseparable, and the two can be transformed into each other under certain conditions. Jeong et al. [26] designed a new type of microrobot without complicated mechanical parts. Two actuation schemes were proposed. The first is the electromagnetic actuation of microrobots, which is based on a pair of Helmholtz and Maxwell electric coils. The second is acoustic bubble actuation for drug release. When two columnar bubbles of different lengths are simultaneously exposed to sound waves, only the bubble whose natural frequency corresponds to the applied frequency vibrates and causes a microflow, while the other bubble remains motionless. Experiments show that the microrobot can be propelled to a desired position through electromagnetic actuation, and carry, release, and inject the drug. Based on this, the new microrobot is expected to be used in targeted drug delivery and other biomedical fields.

Si et al. [27] proposed a theoretical concept of a nanorobot consisting of a nanoparticle and four single-stranded DNAs placed on a quad-nanopore device for motion control. When an electric field is applied, the four single-stranded DNAs of the robot can be captured one by one by the nanopores. Studies have shown that electroosmosis and electrophoresis are the main methods to actuate robot movement. By changing the charge on the nanopores, the strength and direction of electroosmosis can be well controlled, so as to make the robot move along the surface of a graphene film in a desired trajectory. This study provided a lot of useful insight in the design of nanorobots. In the future, such designed nanorobots could be integrated into a silicon-based chip to perform specific actions. Also they could be integrated into a solid-state nanopore platform to capture and sense target molecules. This shows that the application prospects of nanorobots are broad. However, the difficulty of how to achieve the accurate control of direction and speed of motion cannot be ignored.

Qu et al. [28], who also wanted to study electromagnetic fields in the application of micro/nanorobots, used finite element methods to simulate the magnetic field generated by a coil assembly in order to actuate micro/nanorobots more effectively. At the same time, a three-dimensional magnetic simulation was carried out to reveal the magnitude of the magnetic force on a cylindrical micro/nanorobot. Via experimental measurements, the simulation results were verified and the validity of the simulation was proved. The results of this research provide reference values for the application of electromagnetically actuated micro/nanorobots.

Introducing conductive materials into a micro/nanorobot and then adjusting the surface charge of the robot or the electrochemical reaction on the interface through electric fields can also yield actuation. Zhang et al. [29] proposed an interdigital microelectrode system. When an AC electric field is applied, metal electrolyte spherical micro/nanorobots constrained by hydrodynamics can be polarized along the center line of the electrode, and the movement speed can be controlled. In addition, the micro/nanorobots moving in the same direction move in single file, while the micro/nanorobots moving in the opposite direction mainly reorient and move with each other. At high particle density, as the multibody interaction becomes more complex, turbulent aggregates are formed.

Guo et al. [30] proposed a method to manipulate nanomotors by using a three-dimensional orthogonal microelectrode device to apply AC and DC electric fields, which can accurately control the transport of cargo. The DC electric field changes the transmission speed through electrophoresis and electroosmosis effects, and the AC electric field independently and accurately guides the nanomotor through electric torque on the dipole. The effective combination of the two enables loading, transportation, and release of cargo. The proposal of this research further promotes high-precision adjustment and real-time velocity control of nanomotors. This is a key step to realize the application of nanofactories and nanorobots in the future.

#### Light field actuation

Wang et al. [31] designed a needle-shaped liquid metal gallium nanoswimmer with controllable movement under near-infrared laser irradiation. Its propulsion force is mainly derived from the thermophoresis force generated by the temperature gradient along the longitudinal axis. Experiments show that the speed of the nanoswimmer can be adjusted by the light intensity. Under a laser intensity of 5 W·cm^−2^, the nanoswimmer can reach a speed of 31.22 μm/s. The potential of this nanoswimmer in biomedical applications and active soft materials provides support for future research.

Sato et al. [32] designed a molecular robot similar to an amoeba, innovatively using specific photoresponsive DNA signaling molecules to control the movement of the robot. Unlike other nanorobots, this robot is entirely composed of biological and chemical components. The main structure is a vesicle composed of phospholipid bilayers, which also contains an actuator and a clutch inside. When the robot is irradiated by ultraviolet light, the photoresponsive DNA will split into single strands and attach to the microtubules. The slide of microtubules causes the outer cell membrane to change shape, which transforms the robot from an inactive sphere to an active moving non-sphere. Conversely, when the robot is illuminated by visible light, the microtubes cannot interact with the membrane, that is to say, the clutch is disengaged, and the robot will also change back to a spherical shape and stop moving. This research successfully simulated the movement of cells and provided a powerful support platform for the development of molecular robots. But there are still some limitations. For example, the motion of the robot has no directionality and the behavior conversion process is not reversible. This will become a key concern in future research.

Miskin et al. [33] researched a new type of electrochemical actuator, which is compatible with existing silicon electronic devices, has a controllable voltage, and can wake up a robot to move. For a long time, the lack of micro/nanoscale actuators has limited the development of micro/nanorobots. Traditional piezoelectric actuators are suitable for millimeter-sized robots, but not for the micro/nanoscale. Therefore, the innovation was to design a new type of electrochemical actuator and to use it as legs of the robot. It was made of nanoscale platinum and manufactured by a standard photolithography process. When the actuator is stimulated by laser pulses, it will bend and make the robot walk. The research of this technology provides an extremely powerful theoretical basis for the manufacture of actuators in the future.

#### Acoustic field actuation

Ultrasound is sound with a frequency higher than the upper limit of human hearing. The ultrasonic bubble gathering effect generated by external ultrasonic fields or the pressure difference generated by the asymmetric structure of a robot body in an ultrasonic field can enable the robot to move. Wang et al. [34] developed metal rod-shaped micro/nanorobots in batches by template electrodeposition. Under the action of the ultrasonic field, the robots could achieve velocities up to 200 μm/s. Research has shown that standing ultrasonic waves in the megahertz frequency range can suspend, advance, rotate, arrange, and assemble metal microelectrodes in water and solutions of high ionic strength. When the ultrasonic frequency is tuned to generate a vertical standing wave, the metal rod is suspended in the midpoint plane of a cylindrical cell. A rapid axial movement of 200 μm/s of the metal microprobe at the resonance frequency was observed using continuous or pulsed ultrasound. In addition, the motion of the metal rod actuated by ultrasound is not sensitive to the addition of salt in the solution, which makes it possible to use ultrasound to actuate and control metal micro/nanorobots in biological media.

Lee et al. [35] proposed a new ultrasonic actuation mechanism. By generating an acoustic radiation force in a standing wave to capture and move particles, it was possible to realize drug particle/cell manipulation and micro/nanorobot actuation in clinical biology and medicine. An ultrasonic transducer and an electric linear platform were used, which could control the driving speed and movement of the particles along a desired trajectory by changing the frequency range of the transducer and the scanning time and other parameters. Although this concept has limitations such as being interfered by media in actual body tissues, it provides the possibility to apply accurate drug delivery in peripheral blood vessels.

Valdez‐Garduño et al. [36] proposed a new method that uses an external magnetic field to fix the direction of a micromotor and converts the ultrasound-induced oscillation motion of a Janus microstructure with asymmetric density into a translational motion. The acoustic movement of the Janus microstructure is based on the selection of materials with different densities and shapes. Experiments show that density asymmetry causes uneven oscillations and generates a flow at the boundary. Geometric asymmetry enhances the flow and provides a driving force for translational motion. In addition to ultrasonic driving, the Janus microstructure in this method can also be propelled by magnetic fields or chemical driving. This research provides a new option for the design and application of ultrasonic propulsion micro/nanomotors.

### Self-actuation

#### Chemical self-actuation

The essence of chemical self-actuation is to construct an asymmetric field, such as bubble recoil or concentration gradients to break the static balance of a micro/nanorobot and make it move. Whitesides et al. [37] first designed a micro/nanoscale self-actuation robot in 2002. The terminal of this micro/nanorobot was equipped with a platinum-plated porous glass filter, which can generate a large amount of oxygen through decomposition of H_2_O_2_ on the platinum surface and generate an actuation force at the interface between H_2_O_2_ solution and air to push the robot. This process converts the chemical energy in the surrounding environment into kinetic energy through an oxidation–reduction reaction. Although this design has some shortcomings, as the earliest self-actuation micro/nanorobot, it provided inspiration and ideas for subsequent research.

On the basis of previous research, Bao et al. [38] innovatively designed a V-shaped micro/nanorobot made of platinum only. The nanorobot is propelled by oxygen molecules generated by the decomposition of H_2_O_2_ and can maintain a rotating motion and navigate in a stable trajectory. Compared with previous studies, the manufacturing process of the micro/nanorobot is simple and clockwise and counterclockwise bidirectional movement were achieved, instead of the one-way movement only that traditional micro/nanorobots can perform. Therefore, the success of this research can be said to be a big step forward for micro/nanorobots.

Chen et al. [39] proposed Z-shaped Au/Pt hybrid self-actuation micro/nanorobot for cancer treatment and targeted drug delivery systems. It is based on self-electrophoretic actuation. The platinum end is designed to be wider than the gold end. Thus, the movement of the micro/nanorobot was directed towards the platinum end, and a maximum movement speed of 4 μm/s was achieved. After that, Chen et al. [40] designed a Z-shaped platinum hybrid nanorobot in order to meet the growing demand for micro/nanorobots in the biomedical field. It was manufactured using a combination of focused ion beam and plasma sputtering, and the actuation mechanism of the robot is self-electrophoretic. Because the shape of the micro/nanorobot was different, the Z-shaped micro/nanorobot can use two opposite forces on different sides to actuate rotation. In addition, new research has found that the measured actuation force is of the order of 10^−14^ N and is related to the concentration of H_2_O_2_. This research provides vital insights and theoretical foundations for in-depth exploration of the performance of micro/nanorobots in the future.

Xu et al. [41] developed a micro/nanorobot based on platinum catalysis, which can be actuated by an electric mechanism decomposing H_2_O_2_. Xu used a chemical vapor deposition method to obtain a large number of helical molecular chains and studied the motion behavior related to the number of rotations. With increasing number of rotations, the micro/nanorobot may counter greater resistance during motion. In addition, the study also found that the concentration of H_2_O_2_ and the distribution of platinum may also affect the movement. Although chemical vapor deposition offers a high yield, the oxide produced during chemical vapor deposition reduces the effective controllability, which is a new perspective of the coming research. In the future, obtaining a large number of controllable spiral motors needs to be focused on.

**Figure 2:**
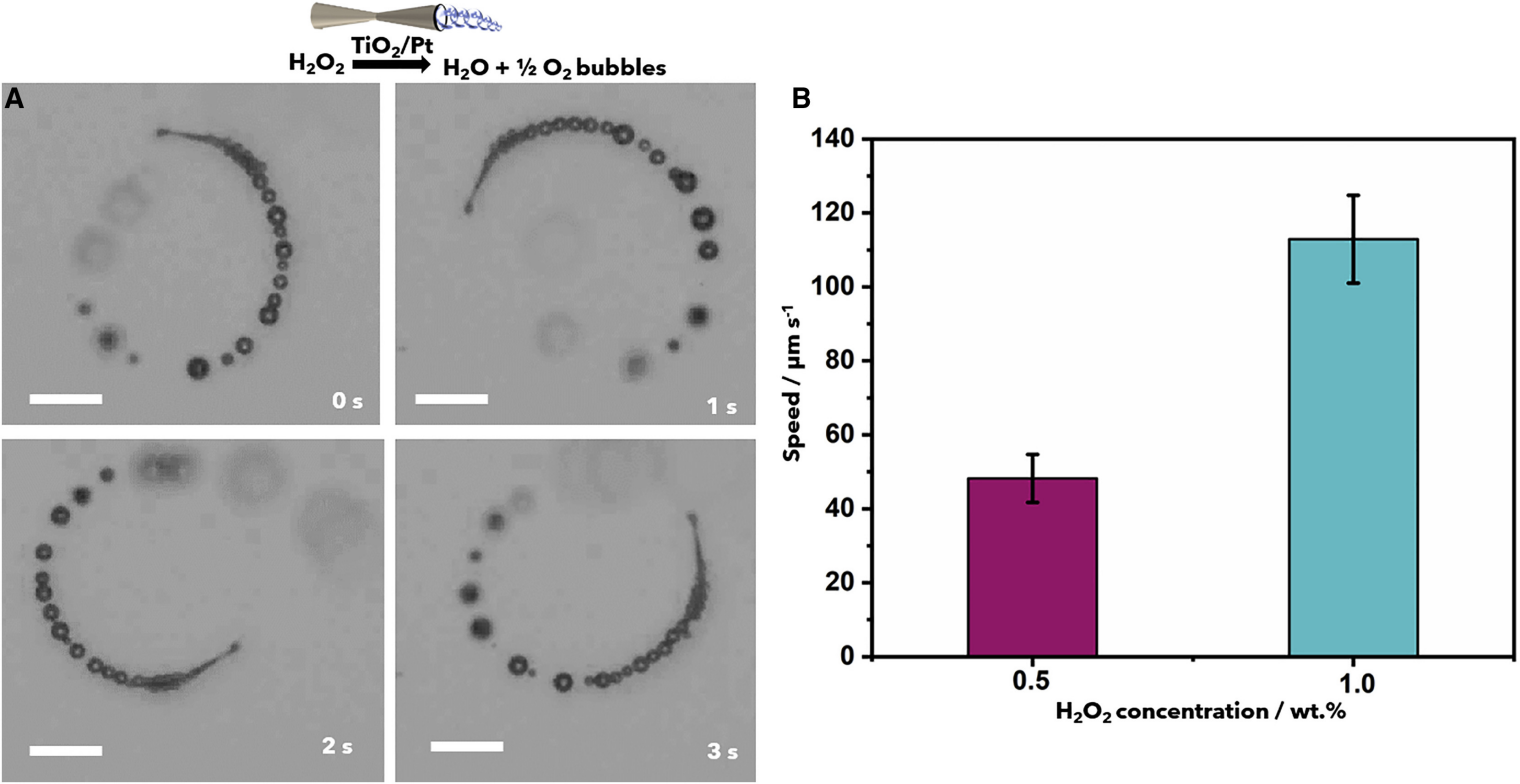
Movement of TiO_2_/Pt microrobots. (A) Snapshot images of a TiO_2_/Pt microrobot moving in a solution of 0.5 wt % H_2_O_2_ and 0.1 wt % surfactant. Scale bars: 10 μm. (B) Speed of the TiO_2_/Pt microrobots at different concentrations of H_2_O_2_ (*n* = 10; error bars represent the standard deviation). Reproduced from [42], Copyright © 2020 Villa et al., licensed under a Creative Commons Attribution-NonCommercial-NoDerivatives 4.0 International License (CC BY-NC-ND 4.0), https://creativecommons.org/licenses/by-nc-nd/4.0/. This content is not subject to CC BY 4.0.

Villa et al. [42] designed a self-actuated tubular microrobot, which can effectively deal with dental plaque and other oral problems through the combination of microbubbles and reactive substances formed on the surface of the biofilm. The robot is composed of biocompatible materials, and the internal structure is decorated with platinum nanoparticles, which can decompose H_2_O_2_ fuel into bubbles. The characteristics of the tubular microrobot are illustrated in Figure 2. The bubbles are discharged from one end of the tube, triggering it to move in a circular trajectory. Maximum velocities of 48 ± 6 and 113 ± 12 µm/s have been reached at fuel concentrations of 0.5 and 1 wt %, respectively. Experiments show that this method can yield more than 95% of dental plaque removal within 5 min, which is currently difficult to achieve with other traditional methods. This research is an innovation in the field of dental surgery. Due to the autonomous motion and the tiny size of the microrobots, it is reasonable to believe that it will also have a significant impact in other medical fields besides oral treatment.

Inspired by the molecular motion of proteins in animal cells, scientists integrated enzymes as catalytic engines, using chemical reactions to provide power for micro/nanorobots. Compared with traditional chemical self-actuation methods, enzyme actuation uses biocompatible fuels and has a better development prospect.

Patino et al. [43] combined DNA nanoswitches with urease-powered micromotors to achieve the goal of swimming and sensing the pH value of the environment. In this study, the urease-powered micromotor served two functions. It detected changes in the pH value through FRET imaging while propelling the switches. The entire self-actuation process is realized by urease-mediated conversion of urea into ammonia and carbon dioxide. Unlike traditional nanoprobes, DNA nanoswitches can perform pH value measurements within a few microseconds. This research is a big step towards real-time monitoring of microenvironmental changes and the internal activities of micromotors during swimming. Also, it provides a new idea for the future use of micromotors to monitor the health of human cells or tissues.

Wang et al. [44] designed a nanomotor based on lipase. Here, lipase provides two functions. The first is as a power engine using triacetin as fuel and achieving particle diffusion through catalytic reaction with it. The second is an active cleaning function that can degrade triglyceride droplets by about 98% within 50 min. This shows great potential for biomedical applications and can be used to treat triglyceride-related diseases. In the future, the degradation of other substances could be explored, that is, the removal of thrombi or oil spills.

#### Biological self-actuation

Inspired by nature, scientists choose appropriate biological materials as actuators of micro/nanorobots, for example, the flagella or cilia of swimming microorganisms. Behkam and Sitti [45] suspended droplets of bacteria, such as E. coli, and polystyrene in a solution of water and glucose. After absorbing the glucose nutrient, the rotating flagella of the bacteria pushed the droplets forward, and then copper sulfate was added to make the bacterial flagella enter a “paralysis” state. Finally, ethylenediaminetetraacetic acid was used to separate the copper sulfate, so that the bacteria could move again. The success of this research created a theoretical basis for the use of biomaterials for self-actuation.

Magdanz et al. [46] proposed to use spermatozoa containing flagella as an actuation force for the preparation of micro/nanorobots. When combining sperm cells with nanotubes, the swing of the sperm flagella interacts with the microtubes to actuate the movement of the robot. Due to the high motility and the safety advantages of spermatozoa compared with bacteria and other microorganisms, sperm-driven micro/nanorobots are expected to become a promising device in the field of artificial insemination and other reproductive technologies.

Martel [47] found that, in previous studies, there was no content about MC-1 magnetotactic bacteria (MTB) in the treatment of cancer, especially as a supplementary means to advance in smaller capillaries. Therefore, Martel innovatively used MC-1 magnetotactic bacteria combined with ferromagnetic materials to push microbeads and therapeutic agents together for targeted therapy. This research result indicated that the use of magnetotactic bacteria is an effective method to treat cancer, and its application urgently needs more scholars to explore.

Xing et al. [48] used marine-derived magnetotactic bacteria (AMB-1) as a template to build an intelligent micro/nanorobot called AI microrobot. Through magnetic/optical sequential manipulation, magnetically controlled navigation was realized in mice. Under a magnetic field, precise movement control at the micrometer scale of a single robot or a group of robots was achieved, with real-time tracking in the body through dual-mode fluorescence and magnetic resonance imaging. Using magnetic targeting, the micro/nanorobot broke through complex physiological barriers and entered tumors while carrying a photosensitizer. After that, local high temperature was generated by a near-infrared laser, and observable and accurate treatment of the tumor was realized. At present, this technology is at the leading level in the world and will point the way for future research on micro/nanorobots with autonomous actuation.

## Conclusion

This article introduces the latest research progress of micro/nanorobots regarding the actuation mechanism. The advantages and disadvantages of various actuation methods are summarized in Table 1.

**Table 1:** Comparison of actuation methods for micro/nanorobots.

Type of actuation	Actuation method	Advantages	Disadvantages

external field	magnetic field	strong penetrating power, no damage to living biological systems	safety problems concerning the application of high-intensity magnetic fields in biomedicine
	electric field	strong penetrating power, adjustable electric field intensity	limitations on biomedical applications of electrodes
	light field	precise targeting	easily causes irreversible damage to biological materials, harmful UV light
	acoustic field	strong penetrating power and actuation force, height adjustable, biocompatibility within a certain frequency range	easily causes damage to biological tissues

self-actuation	chemical	good biocompatibility	fuel safety issues, noxious H_2_O_2_, nontoxic urea, short action time, lack of feedback
	biological	high safety	need to maintain activity, limited range

In the past few years, the field of micro/nanorobots has developed rapidly. Currently, there are still many issues about micro/nanorobots that need to be studied urgently. The following trends in the development of micro/nanorobots can be foreseen. First, the study of hybrid actuation modes. Although each actuation mode is listed separately in this paper, there have been many combined actuation modes [49–50]. A single control method has advantages and disadvantages, while hybrid actuation methods can make use of the advantages and circumvent the disadvantages. Therefore, it is reasonable to believe that the combined use of different actuation modes may become a trend in the future research. Second, the study of motion control of micro/nanorobots. It is not sufficient to pay attention to the actuation of micro/nanorobots. While exploring actuation technologies, motion control methods, such as magnetic field control, ultrasonic control, photoelectric control, and chemical reaction control, should be promoted at the same time in order to facilitate real-time monitoring of the working conditions of micro/nanorobots and to achieve optimal control. Third, the study of fabrication and new materials of micro/nanorobots. With the continuous development of nanotechnology, more and more new materials and new technologies have emerged [51–52]. In order to make good use of micro/nanorobots in biomedicine and other fields, new fabrication methods of micro/nanorobots should be explored, and more biocompatible nanomaterials should be selected to reduce costs and expand applications. Finally, problems of clinical testing and practical application. There is still a long way to go before the micro/nanorobots are put into clinical testing and practical application, and this will face great challenges. With the continuous improvement of various technologies, more and more researchers will be attracted to join the field of micro/nanorobots for exploration and application in the future.

## References

[1] Wu Z, Lin X, Zou X, Sun J, He Q (2015). ACS Appl Mater Interfaces.

[2] Ma X, Wang X, Hahn K, Sánchez S (2016). ACS Nano.

[3] Hoop M, Mushtaq F, Hurter C, Chen X-Z, Nelson B J, Pané S (2016). Nanoscale.

[4] Sun M, Fan X, Meng X, Song J, Chen W, Sun L, Xie H (2019). Nanoscale.

[5] Rosli N F, Mayorga-Martinez C C, Fisher A C, Alduhaish O, Webster R D, Pumera M (2020). Appl Mater Today.

[6] Thubagere A J, Li W, Johnson R F, Chen Z, Doroudi S, Lee Y L, Izatt G, Wittman S, Srinivas N, Woods D (2017). Science.

[7] Feng L, Di P, Arai F (2016). Int J Rob Res.

[8] Yang L, Zhao Y, Xu X, Xu K, Zhang M, Huang K, Kang H, Lin H-c, Yang Y, Han D (2020). Angew Chem, Int Ed.

[9] Liang C, Zhan C, Zeng F, Xu D, Wang Y, Zhao W, Zhang J, Guo J, Feng H, Ma X (2018). ACS Appl Mater Interfaces.

[10] Liu J, Li J, Wang G, Yang W, Yang J, Liu Y (2019). J Colloid Interface Sci.

[11] Ren M, Guo W, Guo H, Ren X (2019). ACS Appl Mater Interfaces.

[12] Feynman R P (2011). Resonance.

[13] Grifantini K (2019). IEEE Pulse.

[14] Abbott J J, Peyer K E, Lagomarsino M C, Zhang L, Dong L, Kaliakatsos I K, Nelson B J (2009). Int J Rob Res.

[15] Walker D, Kübler M, Morozov K I, Fischer P, Leshansky A M (2015). Nano Lett.

[16] Chen X Z, Hoop M, Mushtaq F, Siringil E, Hu C, Nelson B J, Pané S (2017). Appl Mater Today.

[17] Dreyfus R, Baudry J, Roper M L, Fermigier M, Stone H A, Bibette J (2005). Nature.

[18] Steager E B, Selman Sakar M, Magee C, Kennedy M, Cowley A, Kumar V (2013). Int J Rob Res.

[19] Li T, Li J, Zhang H, Chang X, Song W, Hu Y, Shao G, Sandraz E, Zhang G, Li L (2016). Small.

[20] Li T, Li J, Morozov K I, Wu Z, Xu T, Rozen I, Leshansky A M, Li L, Wang J (2017). Nano Lett.

[21] Mushtaq F, Torlakcik H, Hoop M, Jang B, Carlson F, Grunow T, Läubli N, Ferreira A, Chen X-Z, Nelson B J (2019). Adv Funct Mater.

[22] Wang X, Ho C, Tsatskis Y, Law J, Zhang Z, Zhu M, Dai C, Wang F, Tan M, Hopyan S (2019). Sci Rob.

[23] Kim E, Jeon S, An H-K, Kianpour M, Yu S-W, Kim J-y, Rah J-C, Choi H (2020). Sci Adv.

[24] Hsu A, Zhao H, Gaudreault M, Foy A W, Pelrine R (2020). IEEE Rob Autom Lett.

[25] Zheng Z, Wang H, Dong L, Shi Q, Li J, Sun T, Huang Q, Fukuda T (2021). Nat Commun.

[26] Jeong J, Jang D, Kim D, Lee D, Chung S K (2020). Sens Actuators, A.

[27] Si W, Yu M, Wu G, Chen C, Sha J, Zhang Y, Chen Y (2020). ACS Nano.

[28] Qu C, Pei Y-C, Xu L, Xia Z-R, Xin Q-Y (2020). Prog Electromagn Res M.

[29] Zhang L, Xiao Z, Chen X, Chen J, Wang W (2019). ACS Nano.

[30] Guo J, Gallegos J J, Tom A R, Fan D (2018). ACS Nano.

[31] Wang D, Gao C, Si T, Li Z, Guo B, He Q (2021). Colloids Surf, A.

[32] Sato Y, Hiratsuka Y, Kawamata I, Murata S, Nomura S-i M (2017). Sci Rob.

[33] Miskin M Z, Cortese A J, Dorsey K, Esposito E P, Reynolds M F, Liu Q, Cao M, Muller D A, McEuen P L, Cohen I (2020). Nature.

[34] Wang W, Castro L A, Hoyos M, Mallouk T E (2012). ACS Nano.

[35] Lee H-S, Go G, Choi E, Kang B, Park J-O, Kim C-S (2019). Int J Control, Autom Syst.

[36] Valdez‐Garduño M, Leal‐Estrada M, Oliveros‐Mata E S, Sandoval‐Bojorquez D I, Soto F, Wang J, Garcia‐Gradilla V (2020). Adv Funct Mater.

[37] Ismagilov R F, Schwartz A, Bowden N, Whitesides G M (2002). Angew Chem, Int Ed.

[38] Bao J, Yang Z, Nakajima M, Shen Y, Takeuchi M, Huang Q, Fukuda T (2014). IEEE Trans Rob.

[39] Chen K, Chen T, Liu H, Yang Z (2015). A Pt/Au hybrid self-actuating nanorobot towards to durg delivery system. 10th IEEE International Conference on Nano/Micro Engineered and Molecular Systems.

[40] Chen K, Gu C, Yang Z, Nakajima M, Chen T, Fukuda T (2017). Micromachines.

[41] Xu D, Zhan C, Sun Y, Dong Z, Wang G P, Ma X (2019). Chem – Asian J.

[42] Villa K, Viktorova J, Plutnar J, Ruml T, Hoang L, Pumera M (2020). Cell Rep Phys Sci.

[43] Patino T, Porchetta A, Jannasch A, Lladó A, Stumpp T, Schäffer E, Ricci F, Sánchez S (2019). Nano Lett.

[44] Wang L, Hortelão A C, Huang X, Sánchez S (2019). Angew Chem, Int Ed.

[45] Behkam B, Sitti M (2007). Appl Phys Lett.

[46] Magdanz V, Sanchez S, Schmidt O G (2013). Adv Mater (Weinheim, Ger).

[47] Martel S (2006). Towards MRI-Controlled Ferromagnetic and MC-1 Magnetotactic Bacterial Carriers for Targeted Therapies in Arteriolocapillar Networks Stimulated by Tumoral Angiogenesis. 2006 International Conference of the IEEE Engineering in Medicine and Biology Society.

[48] Xing J, Yin T, Li S, Xu T, Ma A, Chen Z, Luo Y, Lai Z, Lv Y, Pan H (2021). Adv Funct Mater.

[49] Ren L, Zhou D, Mao Z, Xu P, Huang T J, Mallouk T E (2017). ACS Nano.

[50] Jin Z, Nguyen K T, Go G, Kang B, Min H-K, Kim S-J, Kim Y, Li H, Kim C-S, Lee S (2019). Nano Lett.

[51] Guerrero Correa M, Martínez F B, Vidal C P, Streitt C, Escrig J, de Dicastillo C L (2020). Beilstein J Nanotechnol.

[52] Alagarsamy K N, Mathan S, Yan W, Rafieerad A, Sekaran S, Manego H, Dhingra S (2021). Bioact Mater.

